# Evaluation of Speech Outcomes in Children With Cleft Palate Following Primary Single-Stage Palatoplasty

**DOI:** 10.1097/SCS.0000000000012802

**Published:** 2026-04-30

**Authors:** Nina Wijnants, Tim F.P. Ritzen, Maud J.M. Verbeek, Shauni A.D. Caenen-Drummen, Sander M.J. van Kuijk, Darren I. Booi, René R.W.J. van der Hulst, Rutger M. Schols

**Affiliations:** * Department of Plastic, Reconstructive and Hand Surgery, Cleft Team, Maastricht University Medical Centre, MosaKids Children’s Hospital; † Department of Speech and Language Pathology, Cleft Team, Maastricht University Medical Centre, MosaKids Children’s Hospital; ‡ Department of Clinical Epidemiology and Medical Technology Assessment, Maastricht University Medical Centre, Maastricht, The Netherlands; § Department of Plastic, Reconstructive and Aesthetic Surgery, Cleft Team, University Hospital Brussels (UZ Brussel), KidZ Health Castle Children’s Hospital, Free University Brussels (VUB), Brussels Health Campus, Belgium

**Keywords:** Cleft palate, otitis media with effusion, single-stage palatoplasty, speech outcomes, tympanostomy tubes

## Abstract

**Objective:**

To evaluate speech outcomes in children with cleft palate following single-stage closure of the soft and hard palate.

**Design:**

A retrospective cohort study.

**Setting:**

Tertiary cleft care center at Maastricht University Medical Center+ (MUMC+), the Netherlands.

**Patients and Participants:**

A total of 47 children with cleft palate or cleft lip and palate underwent single-stage palatal closure between 2011 and 2018. Children with syndromes associated with significant cognitive or developmental impairment precluding a valid speech assessment were excluded.

**Interventions:**

Primary palatal repair using 2-flap palatoplasty, Furlow double-opposing Z-plasty, or von Langenbeck technique.

**Main Outcome Measure(s):**

Speech was assessed using the Dutch Cleft Speech Evaluation Test (DCSET) and the Dutch version of the Cleft Audit Protocol for Speech—Augmented (CAPS-A). Secondary outcomes included the prevalence of otitis media with effusion (OME) and tympanostomy tube placement.

**Results:**

Speech intelligibility improved significantly at the final assessment (median score 2–1; *P*<0.001). No significant changes were found in voice, hypernasality, hyponasality, nasal emission, nasal turbulence, or grimace. CAPS-A analysis showed resolution of palatalization errors, but increased dentalisation/interdentalisation, likely reflecting temporary compensatory articulation. OME was present in 90.7% of children, and 97.6% required at least 1 tympanostomy tube.

**Conclusion:**

Single-stage palatal closure leads to improved speech intelligibility with stable outcomes across other speech parameters. The high prevalence of middle ear disease underscores the need for multidisciplinary follow-up.

The primary goals of cleft palate repair are to restore functional speech and swallowing and ensure proper function of the velopharyngeal mechanism.^1^ The timing and surgical technique used for palatal closure are known to influence these outcomes.

Historically, the closure of the soft and hard palate was performed in a single surgical procedure. However, concerns regarding its potential impact on maxillofacial growth have led to the adoption of a 2-stage approach, in which closure of the hard palate is delayed until ∼3 years of age.^2^,^3^

Velopharyngeal insufficiency (VPI), a common consequence of cleft palate, can result in hypernasality, nasal emission, nasal turbulence, and reduced intraoral air pressure. These deficits interfere with the production of pressure consonants such as plosives and fricatives.^4^ To compensate, children may adopt alternative articulation strategies, including posterior placements in the pharyngeal or glottal regions and active nasal fricatives, known as compensatory articulations or nonoral cleft-type characteristics.^5^

Speech outcomes in children with cleft palate are frequently assessed using the Cleft Audit Protocol for Speech—Augmented (CAPS-A), a validated and reliable tool utilized by Specialist Cleft Speech and Language Therapists.^6^

The aim of this study was to investigate speech outcomes in children with cleft palate who underwent primary single-stage closure of the soft and hard palate. By using the CAPS-A tool, this study seeks to provide new insights into the effects of this technique on speech development and contribute to the ongoing evaluation of surgical approaches for cleft palate repair.

## METHODS

### Study Design

This retrospective cohort study was conducted at the Department of Plastic, Reconstructive, and Hand Surgery of the Maastricht University Medical Center (Maastricht UMC+, the Netherlands). Patients who presented at the Maastricht UMC+ cleft center between January 2011 and December 2018 and received treatment accordingly were selected. Data were collected to evaluate speech outcomes in children with cleft palate who underwent single-stage closure of the soft and hard palate. In addition, the presence of otitis media with effusion (OME) and the placement of tympanostomy tubes were recorded to provide insight into a potential relationship between otological disease and speech development.

### Study Population

Patients were eligible for inclusion if they had a diagnosed cleft palate (of any type), demonstrated adequate cognitive or developmental capacity to participate in speech assessment, and were registered with the cleft team at Maastricht UMC+ within 6 months after birth. Children with a syndromic cleft palate were only excluded if the associated syndrome involved significant cognitive or developmental impairment that precluded a valid speech assessment.

### Outcome Measures

The primary outcome was postoperative speech outcomes using the Dutch Cleft Speech Evaluation Test (DCSET),^7^ and the CAPS-A in the Dutch language (based on the original CAPS-A developed by Sell, Harding-Bell, and Sweeney, 2006). The secondary outcomes included the presence of OME and the number of tympanostomy tube placements per patient.

#### Speech Outcome Scoring (CAPS-A Dutch)

Postoperative speech outcomes were scored using the Dutch version of the Cleft Audit Protocol for Speech—Augmented (CAPS-A Dutch). Intelligibility was rated on a 0 to 4 scale, where 0 indicates speech is intelligible, and 4 indicates speech is unintelligible. Voice was scored as either absent (0) or present (1). Hypernasality was evaluated on a 0 to 4 scale, with 0 indicating no hypernasality and 4 representing severe hypernasality during the production of vowels and voiced consonants. Hyponasality was scored on a 0 to 2 scale, where 0 indicates absence, and 2 indicates marked hyponasality. Nasal emission and nasal turbulence were each scored on a 0 to 2 scale, with 0 indicating absence and 2 indicating frequent occurrence in >10% of oral pressure consonants. Finally, grimace was scored as absent (0) or present (1).

### Data Collection

We collected demographics, cleft classification, surgical details, speech therapy and assessment, and otological history, as described in detail below.

#### Demographics

Demographic data were extracted from medical records and included the patient’s sex and age at the time of primary surgery. In addition, syndromic status and family history of cleft were recorded.

#### Cleft Classification

Cleft type was classified according to the Veau classification system: soft palate only (Veau I), soft and hard palate (Veau II), unilateral cleft lip and palate (Veau III), or bilateral cleft lip and palate (Veau IV).

#### Surgical Treatment

All patients underwent primary single-stage closure of the soft and hard palate. Three surgical techniques were used: Furlow double-opposing Z-plasty, Bardach 2-flap palatoplasty, and Von Langenbeck palatoplasty. The choice of technique was based on cleft anatomy and the surgeon’s clinical judgment. All procedures were performed by 1 of 2 experienced plastic surgeons specializing in cleft care, working at Maastricht University Medical Center+.

#### Speech Therapy and Assessment

Postoperative speech therapy was provided by extramural speech therapists to whom patients were referred when indicated by a specialized cleft speech therapist of the cleft team. Therapy was typically initiated on a weekly basis and gradually reduced in frequency toward the end of treatment, as advised by the evaluating cleft speech therapist in collaboration with the treating therapist. The therapy primarily focused on phonetic cleft speech characteristics and velopharyngeal insufficiency (VPI); if phonological speech disorders were present, these were also addressed.

Due to the young age at which patients underwent surgery, no preoperative speech assessments were performed. The first postoperative perceptual speech assessment was conducted by experienced cleft speech therapists using the DCSET, a standardized protocol specifically designed for children with orofacial clefts and implemented in all Dutch cleft centers. The results of this assessment were extracted from the patients’ medical files and then retrospectively scored by 2 trained cleft speech therapists (M.V. and S.C.) using the CAPS-A Dutch.

Final postoperative perceptual assessments were performed live by one of the same trained speech therapists (M.V. and S.C.), again using the CAPS-A protocol. To evaluate inter-rater reliability, each therapist reassessed 50% of the patients previously evaluated live by the other, based on the same speech recordings. The scores for intelligibility, voice, resonance, nasal emissions, and nasal turbulence were compared to determine the IRR.

All speech therapists applying this protocol had specialized training, were certified by the Speech Pathology Working Group of the Dutch Association for Cleft Palate and Craniofacial Anomalies (NVSCA), and participated in national calibration sessions to maintain that certification. VPI was diagnosed through clinical examination, which included assessment of soft palate length and mobility, as well as the child’s ability to follow instructions. When perceptual speech assessment revealed signs suggestive of velopharyngeal insufficiency, such as hypernasality or nasal air emission, additional diagnostic modalities, including nasometry, mirror tests, nasoendoscopy, or videofluoroscopy, were performed to objectively assess velopharyngeal function and confirm the diagnosis.

Nasometry was performed using the Nasometer II model 6450 (Kaypentax, Montvale, NJ). During the nasometry tests, patients were asked to read aloud validated nasal, oronasal, and denasal sentences in Dutch. In the Dutch language, a nasality score >2SD or <2SD is considered abnormal.

While patients were repeating several oral sounds and/or sentences aloud after the speech therapist, the presence of nasal emissions was objectified by mirror tests. For each nostril, the presence of nasal emissions and whether these emissions were audible was noted.

For the analysis of the results of the CAPS-A, Dutch cleft-type speech characteristics (CTCs) were merged into 4 clusters of anterior oral, posterior oral, nonoral, and passive characteristics.

#### Otological History

Medical records were screened for a history of otitis media and the placement of ventilation tubes.

### Ethical Considerations

Approval for this study was obtained from the Institutional Review Board of Maastricht University Medical Center (Maastricht UMC+, the Netherlands, METC 2024-0524).

### Data Analysis

Descriptive statistics for patient characteristics were reported as absolute numbers and percentages, or as means and SDs.

To assess the reliability of the perceptual speech assessments, inter-rater reliability was evaluated between the independent assessments of 2 experienced speech therapists. For the ordinal CAPS-A Dutch variables, the intraclass correlation coefficient (ICC) was calculated. For the categorical CAPS-A Dutch variables, absolute agreement and the Cohen κ coefficient were used.

Ordinal outcomes derived from the CAPS-A Dutch were compared over time points using the Wilcoxon signed-rank test. For binary outcomes, the McNemar test was used. Cleft speech characteristics, evaluated as part of the primary outcome, were analyzed descriptively and presented in tabular form. Nonparametric Kruskal-Wallis rank-sum tests were used as an exploratory analysis to compare median changes in speech outcome scores between surgical techniques, as cumulative link mixed model (CLMM) analyses could not reliably estimate group effects. The presence of OME and tympanostomy tube placement was described narratively without formal statistical analysis.

## RESULTS

An initial review of the local cleft database identified 47 children with cleft palate or cleft lip and palate who underwent primary single-stage closure of both the soft and hard palate and were enrolled in this study. Of these, 4 patients were excluded from the analysis of the secondary outcomes because their otological care was provided at an external hospital, precluding consistent follow-up. Consequently, all 47 patients were included in the analysis of the primary outcome, while 43 patients were analyzed for the secondary outcomes. This study population comprised 23 boys (49%) and 24 girls (51%), with a mean age of 10 months at the time of cleft palate repair. Among these patients, 25 had an isolated cleft palate (Veau I or II), and 22 had cleft lip involvement (Veau III or IV), all of whom had previously undergone lip closure before palatal repair. The Bardach 2-flap palatoplasty technique was used in 30 patients, the Furlow double-opposing Z-plasty in 15 patients, and the Von Langenbeck technique in 2 patients. Several patients presented with associated syndromes, the distribution of which is summarized in Supplemental Table 1, Supplemental Digital Content 1, http://links.lww.com/SCS/J446.

### Speech Therapy

Of the 47 patients included in the study, 27 received postoperative speech therapy, whereas 20 did not. Speech therapy was initiated when speech assessments performed by specialized cleft speech therapists identified articulation errors, resonance problems, or other cleft-related speech characteristics requiring intervention. It was provided by dedicated cleft speech therapists according to national cleft care guidelines.

### Inter-Rater Reliability

Inter-rater reliability for the sum scores of the perceptual speech assessment between live and recorded playback evaluations was represented by an ICC of 0.99 (95% CI: 0.98–1.00) between the 2 cleft speech therapists (M.V. and S.C.). For the separate scores of the CAPS-A Dutch, the voice parameter in particular showed complete agreement, resulting in both an absolute agreement and a Cohen κ of 1.00.

### Primary Outcome: Postoperative Speech Outcomes (CAPS-A Dutch)

The interval between the first and final speech assessments varied across patients, with a mean duration of 5.6±2.3 years (range: 4 mo–11.2 y).

Speech intelligibility was rated on a 0 to 4 scale, where 0 indicates speech is intelligible and normal, and 4 indicates speech is unintelligible. The median intelligibility score improved from 2 at the first assessment (IQR: 1–2) to 1 at the final assessment (IQR: 0–1), with a *P*<0.001.

Voice was scored as either 0 (absent) or 1 (present). An abnormal voice was observed in 8.5% of patients at the first assessment, decreasing to 4.3% at the final assessment (95% CI: 0.5–14.5). The McNemar test showed no statistically significant difference between first and final assessments (*P*=0.68).

Hypernasality was scored on a scale from 0 to 4, where 0 indicates the absence of hypernasality and 4 represents severe hypernasality during the production of vowels and voiced consonants. The median hypernasality score was 0 at both the first and final assessments (IQR: 0–2 for both). This difference was not statistically significant (*P*=0.24).

At the first assessment, 22 out of 47 children (46.8%) presented with hypernasality, with a subgroup median score of 2 (IQR: 2–3). At the final assessment, 19 out of 47 children (40.4%) demonstrated hypernasality, with a subgroup median score of 2 (IQR: 2–3).

Hyponasality was scored on a scale from 0 to 2, where 0 indicates the absence of hyponasality and 2 represents marked hyponasality. The median hyponasality score was 0 at both the first and final assessments (IQR: 0–0 for both). This difference was not statistically significant (*P*=0.77).

Nasal emission was scored on a scale from 0 to 2, where 0 indicates the absence of nasal emission and 2 indicates frequent nasal emission occurring in >10% of oral pressure consonants. The median nasal emission score was 0 at both the first and final assessments (IQR: 0–1 for both). This difference was not statistically significant (*P*=0.99).

Nasal turbulence was scored on a scale from 0 to 2, where 0 indicates the absence of nasal turbulence and 2 indicates frequent nasal turbulence present in >10% of oral pressure consonants. The median nasal turbulence score increased from 0 at the first assessment (IQR: 0–1) to 1 at the final assessment (IQR: 0–1). This difference was not statistically significant (*P*=0.55).

Grimace was scored as either 0 (absent) or 1 (present). An abnormal grimace was observed in 6.4% (95% CI: 1.3–17.5) of patients at both the first and final assessments; *P* for difference=1.00.

The change in speech outcomes is illustrated in Figure 1A to E.

**FIGURE 1:**
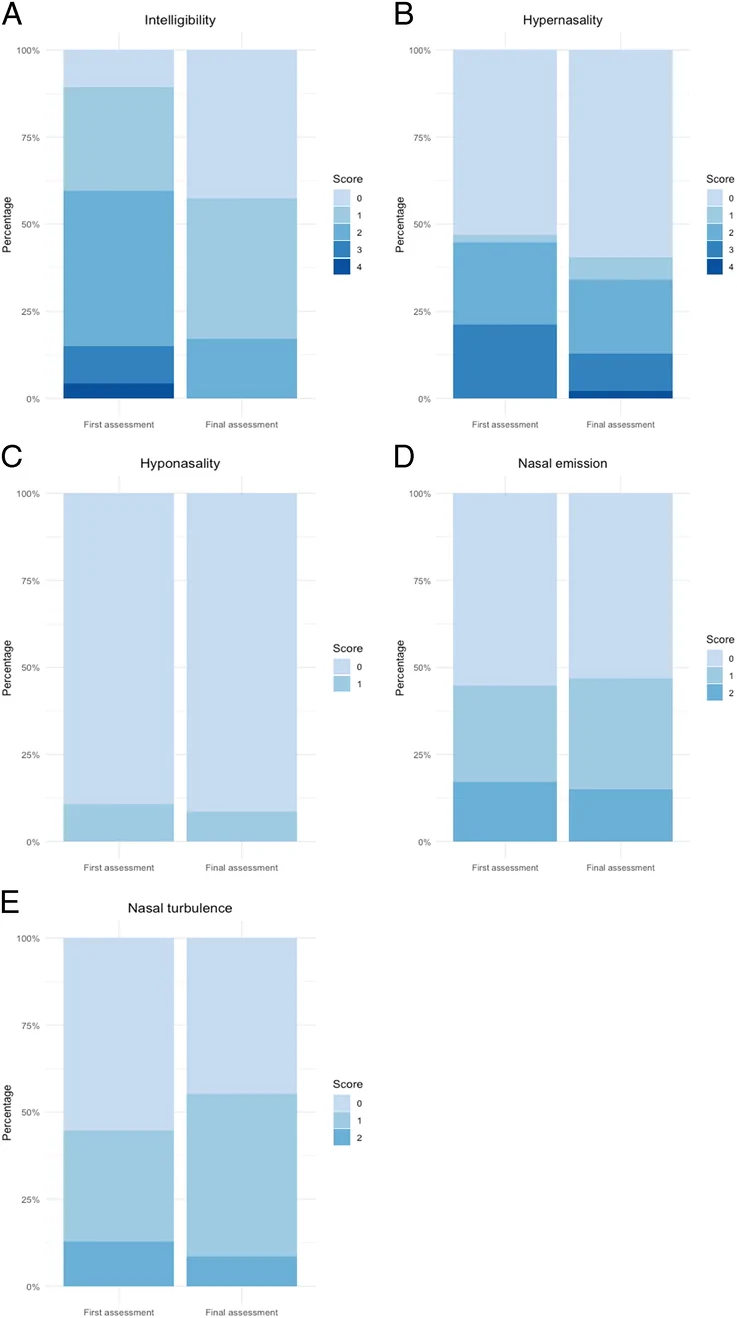
(A–E) Stacked bar charts showing CAPS-A Dutch scores at the first and final assessments for speech intelligibility (A), hypernasality (B), hyponasality (C), nasal emission (D), and nasal turbulence (E).

The Kruskal-Wallis rank-sum test showed median changes in CAPS-A Dutch scores of −1, −1, and −1.5 for Furlow palatoplasty, Bardach 2-flap palatoplasty, and Von Langenbeck palatoplasty, respectively. No statistically significant differences were observed between the surgical techniques for any of the assessed speech outcomes (*P*=0.10–1.00).

### Primary Outcome: Cleft Type Characteristics

Cleft type characteristics were categorized into anterior oral, posterior oral, nonoral, and passive consonant characteristics (Supplemental Table 2, Supplemental Digital Content 2, http://links.lww.com/SCS/J447).

In the category of anterior oral CTCs, the most common error was dentalisation/interdentalisation, which was absent in 25 children (53.2%) at the first assessment and in 11 children (23.4%) at the final assessment. The number of children with 3 or more affected consonants increased from 17 (36.2%) at the first assessment to 35 (74.5%) at the final assessment. Lateralizations remained relatively stable, being absent in 33 children (70.2%) at the first assessment and in 36 children (76.6%) at the final assessment. For palatalizations, an improvement was observed, with absence increasing from 40 children (85.1%) at the first assessment to 47 (100%) at the final assessment.

Regarding posterior oral CTCs, double articulations were absent in all children at both time points. The prevalence of backed-to-velar/uvular articulation decreased, with absence increasing from 35 children (74.5%) at the first assessment to 45 (95.7%) at the final assessment.

In the category of nonoral CTCs, including pharyngeal articulation, glottal articulation, active nasal fricatives, and glottal amplification, most children did not exhibit these errors at either time point. Minor improvements were observed in glottal articulation and active nasal fricatives, with complete resolution at the final assessment.

For passive CTCs, weak and/or nasalized consonants showed a slight increase at the final assessment, with 3 children (6.4%) exhibiting these errors compared with 1 child (2.1%) at the first assessment. In contrast, nasal realization of plosives and/or suspected passive nasal fricatives improved from 3 affected children (6.4%) at the first assessment to none at the final assessment. Gliding of fricatives was absent in all children at both assessments.

### Secondary Outcomes: Frequency of OME and the Placement of Tympanostomy Tubes

OME occurred in 39 out of 43 patients (90.7%). The total number of tympanostomy tube placements ranged from 0 to 7 per patient, with a median of 1 (IQR: 1.0–3.0). A total of 41 patients (97.6%) received at least 1 tympanostomy tube during the follow-up period.

### Additional Surgery During Follow-Up

During follow-up, additional surgery related to velopharyngeal insufficiency or palatal fistula was required in 7 patients (14.9%). Four patients underwent pharyngoplasty for persistent velopharyngeal insufficiency, and 4 patients required surgical closure of a palatal fistula. One patient required both procedures.

## DISCUSSION

In this retrospective cohort study, speech outcomes following primary single-stage closure of both the soft and hard palate were evaluated using standardized assessment tools (DCSET and CAPS-A Dutch), administered by experienced clinicians with excellent inter-rater reliability (ICC=0.99; κ=1.00). We observed a significant improvement in speech intelligibility, with the median score decreasing from 2 to 1 (*P*<0.001). No statistically significant differences in speech outcomes were found between the performed surgical techniques (*P*=0.10–1.00), and no statistically significant changes were found for the other CAPS-A Dutch parameters (voice quality, hypernasality, hyponasality, nasal emission, nasal turbulence, and grimace). However, hypernasality was relatively common within this population: at the first assessment, 46.8% of the children presented with hypernasality, compared with 40.4% at the final assessment. This is congruent with postoperative speech outcomes in recent literature.^8^,^9^ The pattern of findings indicates that the most notable improvement occurred in speech intelligibility, while resonance- and voice-related parameters remained largely stable. This improvement is likely not solely attributable to age-related phonological simplification processes but rather to an interplay of factors, including a stable anatomic foundation following surgical repair, natural speech maturation, and targeted speech therapy. Postoperative speech therapy plays an essential role in this process by helping children functionally utilize their newly reconstructed anatomy and correcting compensatory articulation patterns (if any) at an early stage.^10^ The observed significant improvement in speech intelligibility, combined with the absence of severe articulation errors, supports the previously demonstrated effectiveness of 1-stage palatoplasty in establishing a stable anatomic foundation for speech development and providing an optimal starting point for targeted speech therapy.^11^

Further analysis of the CAPS-A Dutch data showed that palatalization errors were observed in 14.9% of children at the first assessment but were entirely absent at the final assessment, suggesting an improvement in articulation patterns over time, possibly related to speech therapy and functional adaptation following palatal repair. In contrast, anterior oral errors, such as dentalisation and interdentalisation, paradoxically increased between the first and final assessments, as reflected by a reduction in the proportion of children without this error from 53.2% to 23.4%. This increase is most likely related to craniofacial growth patterns. This observation also reflects the ongoing discussion in the literature regarding the potential impact of early single-stage palatal repair on maxillary growth. In particular, the maxilla tends to show restricted growth following early 1-stage palatoplasty, whereas the mandible continues to grow forward, reducing the available space for the tongue to make proper apical contact with the palate.^12^ As a result, the tongue is displaced anteriorly against the incisors, making interdentalisation more prominent over time. This reflects a structural consequence of early closure that often persists until maxillary expansion provides sufficient space for correct tongue placement. These anatomic speech characteristics are more easily corrected through speech therapy and are generally regarded as less detrimental to speech intelligibility and communicative effectiveness than other cleft-related articulation errors.

In contrast, active maladaptive articulation strategies, such as posterior oral, nonoral, and passive CTCs, are typically more detrimental to speech intelligibility. These patterns, however, were already largely absent at the first assessment and showed minimal change over time, suggesting that the surgical intervention was highly effective in preventing or resolving these more severe articulation errors.

For the other cleft-related speech characteristics, the differences between the first and final assessments were modest. Five characteristics showed slight improvement, 2 showed minor deterioration, and 3 remained unchanged due to their complete absence at both time points. This reaffirms the role of targeted speech therapy in refining subtle articulatory deviations and in functionally integrating the reconstructed speech structures.

Despite these generally favorable speech outcomes, additional speech-related surgery during follow-up was required in 7 patients (14.9%). Pharyngoplasty for persistent velopharyngeal insufficiency was performed in 4 patients, and 4 patients required surgical closure of a palatal fistula. These findings are in line with previous studies reporting secondary surgery for velopharyngeal insufficiency in ∼7% to 37% of patients after primary palatoplasty and fistula rates around 8% to 10%.^13^,^14^

Furthermore, OME was highly prevalent in this cohort (90.7%), and nearly all patients (97.6%) required at least 1 tympanostomy tube placement. This high prevalence underscores the well-established association between cleft palate and middle ear dysfunction. Although no formal correlation analysis was performed in this study, previous research suggests that persistent OME may negatively affect speech and language development by causing fluctuating conductive hearing loss, which can disrupt auditory feedback and hinder phonological acquisition.^15^

### Clinical Relevance

Given the high prevalence of speech problems and middle ear dysfunction in children with cleft palate, numerous studies have examined speech outcomes following different surgical protocols and the association between cleft palate and OME.

Recent literature suggests that 1-stage palatal repair can lead to improved speech outcomes. A subgroup meta-analysis comparing 1-stage and 2-stage palatoplasty reported lower rates of VPI and fistula formation in the 1-stage group, suggesting that single-stage closure may achieve adequate anatomic and functional reconstruction in a single procedure.^16^ Another meta-analysis found reduced VPI rates at 5 years of age following 1-stage closure compared with delayed hard palate repair (9% versus 24%), although the certainty of evidence was rated as very low due to small sample sizes and potential reporting bias.^17^

In addition, our findings confirm the ongoing otological burden in this population. We observed a prevalence of OME of 90.7%, with 97.6% of patients requiring tympanostomy tube insertion, figures that are consistent with previous studies reporting OME in ∼90% of children with cleft palate^18^,^19^,^20^ and tube placement in up to 98%.^20^,^21^

### Limitations

Several limitations should be acknowledged. The retrospective nature of this study made it dependent on the accuracy and completeness of medical records, which limited not only the ability to control for potential confounding factors but also precluded the consistent inclusion of additional diagnostic tools, such as nasometry and mirror tests. During the first postoperative assessment, speech outcomes were examined live with a perceptual evaluation tool (the DCSET) and, for research purposes, scored with another tool (the CAPS-A in Dutch language) based on medical records. This allows consistent comparison across both time points. Although at the time of this writing, this Dutch version of the CAPS-A is undergoing validation, the CAPS-A has already been validated in other Germanic languages, such as Belgian Dutch,^22^ British English,^6^ and American English.^23^ In contrast to the DCSET, which evaluates speech on the level of words, the CAPS-A evaluates speech in sentences and should therefore be used in children of a minimum of 4 years old. Applying the CAPS-A framework to children as young as 2 years old is therefore not the intended purpose of the protocol. Moreover, the absence of a control group, such as children treated with a 2-stage palatal repair, prevents direct comparison between surgical approaches. The relatively small sample size further reduces the statistical power and limits the generalizability of the findings. Furthermore, the follow-up interval between the first and last assessments varied considerably between patients, resulting in the absence of a uniform timeframe for evaluating speech outcomes. Finally, due to the timing and retrospective nature of the study, it remains unknown whether some patients may develop VPI later in life, as this can occur during craniofacial growth, orthodontic expansion, or orthognathic surgery. Long-term follow-up would therefore be necessary to fully evaluate the stability of speech outcomes after single-stage palatoplasty.^10^

### Future Perspectives

Future research should aim to directly compare single-stage and 2-stage palatal closure to assess not only long-term speech outcomes but also the development of maxillary growth patterns. Since the present study focused exclusively on short-term speech results after single-stage repair, longitudinal studies are needed to provide a more comprehensive understanding of both speech and craniofacial development over time.

## CONCLUSION

Primary single-stage closure of the soft and hard palate represents an effective surgical approach, resulting in significant improvement in speech intelligibility. Other speech parameters remained relatively stable, indicating limited change over time rather than the absence of residual deviations. Continued multidisciplinary follow-up, particularly with targeted speech therapy and proactive otological management, remains essential to achieve optimal functional outcomes.

## Supplementary Material
