# Supplementary material for: Evaluation of Speech Outcomes in Children With Cleft Palate Following Primary Single-Stage Palatoplasty
Source: J Craniofac Surg. 2026 Apr 30;37(11-12):3179–84. doi: 10.1097/SCS.0000000000012802 (PMC13764263; doi:10.1097/SCS.0000000000012802)
Supplement: Supplementary file 1 [file scs-37-03179-s001.docx]

Supplemental table 1: Patient characteristics

| **Characteristic** | **N (%) or mean ± SD** |
| --- | --- |
| Number of patients | 47 |
| **Sex** |  |
| Male | 23 (49%) |
| Female | 24 (51%) |
| Age at time of surgery, months | 10.0 ± 1.5 |
| **Cleft type** |  |
| Veau I | 11 (23%) |
| Veau II | 14 (30%) |
| Veau III | 15 (32%) |
| Veau IV | 7 (15%) |
| **Surgical technique** |  |
| Bardach’s two-flap palatoplasty | 30 (64%) |
| Furlow palatoplasty | 15 (32%) |
| Von Langenbeck palatoplasty | 2 (4%) |
| **Presence of associated syndromes** |  |
| Pierre Robin Sequence | 3 (6%) |
| Stickler syndrome | 2 (4%) |
| Van der Woude syndrome | 1 (2%) |
| Charge syndrome | 1 (2%) |

*SD, standard deviation; N, number*
