# Supplementary material for: Evaluation of Speech Outcomes in Children With Cleft Palate Following Primary Single-Stage Palatoplasty
Source: J Craniofac Surg. 2026 Apr 30;37(11-12):3179–84. doi: 10.1097/SCS.0000000000012802 (PMC13764263; doi:10.1097/SCS.0000000000012802)
Supplement: Supplementary file 2 [file scs-37-03179-s002.docx]

| Supplemental table 2: Change in cleft type speech characteristics | | | |
| --- | --- | --- | --- |
|  |  | **First assessment** | **Final assessment** |
| **Anterior Oral CTCs** | **Dentalisation/interdentalisation** |  |  |
|  | ≤ Two consonants affected | 5 | 1 |
|  | ≤ Three consonants affected | 17 | 35 |
|  | Absent | 25 | 11 |
|  | **Lateralisation** |  |  |
|  | ≤ Two consonants affected | 10 | 3 |
|  | ≤ Three consonants affected | 4 | 8 |
|  | Absent | 33 | 36 |
|  | **Palatalisation** |  |  |
|  | ≤ Two consonants affected | 7 | 0 |
|  | ≤ Three consonants affected | 0 | 0 |
|  | Absent | 40 | 47 |
| **Posterior oral CTCs** | **Double articulation** |  |  |
|  | ≤ Two consonants affected | 0 | 0 |
|  | ≤ Three consonants affected | 0 | 0 |
|  | Absent | 47 | 47 |
|  | **Backed to velar/uvular** |  |  |
|  | ≤ Two consonants affected | 6 | 1 |
|  | ≤ Three consonants affected | 6 | 1 |
|  | Absent | 35 | 45 |
| **Non-oral CTCs** | **Pharyngeal articulation** |  |  |
|  | ≤ Two consonants affected | 0 | 0 |
|  | ≤ Three consonants affected | 0 | 0 |
|  | Absent | 47 | 47 |
|  | **Glottal articulation** |  |  |
|  | ≤ Two consonants affected | 1 | 0 |
|  | ≤ Three consonants affected | 1 | 0 |
|  | Absent | 45 | 47 |
|  | **Active nasal fricatives** |  |  |
|  | ≤ Two consonants affected | 0 | 0 |
|  | ≤ Three consonants affected | 2 | 0 |
|  | Absent | 45 | 47 |
|  | **Glottal amplification** |  |  |
|  | ≤ Two consonants affected | 1 | 0 |
|  | ≤ Three consonants affected | 1 | 0 |
|  | Absent | 45 | 47 |
| **Passive CTCs** | **Weak and/or nasalised consonants** |  |  |
|  | ≤ Two consonants affected | 0 | 1 |
|  | ≤ Three consonants affected | 1 | 2 |
|  | Absent | 46 | 45 |
|  | **Nasal realisation of plosives and/or suspected passive nasal fricatives** |  |  |
|  | ≤ Two consonants affected | 2 | 0 |
|  | ≤ Three consonants affected | 1 | 0 |
|  | Absent | 44 | 47 |
|  | **Gliding of fricatives/affricates** |  |  |
|  | ≤ Two consonants affected | 0 | 0 |
|  | ≤ Three consonants affected | 0 | 0 |
|  | Absent | 47 | 47 |
